# Methyl 4-eth­oxy-2-methyl-2*H*-1,2-benzothia­zine-3-carboxyl­ate 1,1-dioxide

**DOI:** 10.1107/S1600536808021363

**Published:** 2008-07-16

**Authors:** Muhammad Zia-ur-Rehman, Jamil Anwar Choudary, Mark R. J. Elsegood, Noshin Akbar, Hamid Latif Siddiqui

**Affiliations:** a Applied Chemistry Research Centre, PCSIR Laboratories Complex, Lahore 54600, Pakistan; b Institute of Chemistry, University of The Punjab, Lahore 54590, Pakistan; c Chemistry Department, Loughborough University, Loughborough, Leicestershire LE11 3TU, England; d Department of Chemistry, University of Science and Technology, Bannu, Pakistan

## Abstract

In the crystal structure of the title compound, C_13_H_15_NO_5_S, the mol­ecules exhibit weak S=O⋯H—C and C=O⋯H—C inter­molecular inter­actions and arrange themselves into centrosymmetric dimers by means of π–π inter­actions (ring centroids are separated by 3.619 Å, while the closest C⋯C contacts are 3.514 Å). 1,2-Benzothia­zines of this kind have a range of biological activities and are used as medicines in the treatment of inflammation and rheumatoid arthritis.

## Related literature

For related literature on benzothia­zines, see: Ahmad *et al.* (2008 ▶); Bihovsky *et al.* (2004 ▶); Fabiola *et al.* (1998 ▶); Golič *et al.* (1987 ▶); Kojić-Prodić & Rużić-Toroš (1982 ▶); Lombardino *et al.* (1971 ▶); Reck *et al.* (1988 ▶); Zia-ur-Rehman *et al.* (2005 ▶, 2006 ▶, 2007 ▶).
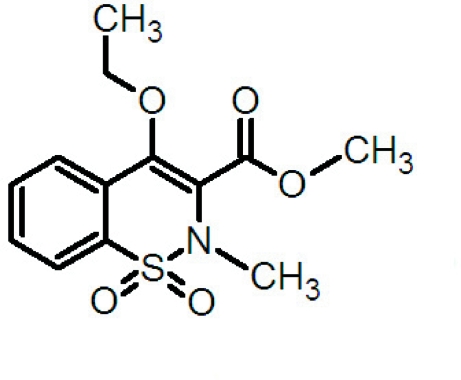


## Experimental

### 

#### Crystal data

C_13_H_15_NO_5_S
*M* *_r_* = 297.32
Triclinic, 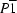

*a* = 7.9810 (4) Å
*b* = 8.1215 (4) Å
*c* = 10.8173 (6) Å
α = 89.4783 (7)°
β = 79.5124 (8)°
γ = 79.3434 (7)°
*V* = 677.33 (6) Å^3^
*Z* = 2
Mo *K*α radiation
μ = 0.26 mm^−1^
*T* = 150 (2) K
0.57 × 0.17 × 0.10 mm

#### Data collection

Bruker APEXII CCD diffractometer
Absorption correction: multi-scan (*SADABS*; Sheldrick, 2007 ▶) *T*_min_ = 0.867, *T*_max_ = 0.975
8153 measured reflections
4069 independent reflections
3678 reflections with *I* > 2σ(*I*)
*R*_int_ = 0.014

#### Refinement

*R*[*F* ^2^ > 2σ(*F* ^2^)] = 0.033
*wR*(*F* ^2^) = 0.096
*S* = 1.07
4069 reflections
184 parameters
H-atom parameters constrained
Δρ_max_ = 0.47 e Å^−3^
Δρ_min_ = −0.34 e Å^−3^

### 

Data collection: *APEX2* (Bruker, 2006 ▶); cell refinement: *SAINT* (Bruker, 2006 ▶); data reduction: *SAINT*; program(s) used to solve structure: *SHELXTL* (Sheldrick, 2008 ▶); program(s) used to refine structure: *SHELXTL*; molecular graphics: *SHELXTL*; software used to prepare material for publication: *SHELXTL* and local programs.

## Supplementary Material

Crystal structure: contains datablocks I, global. DOI: 10.1107/S1600536808021363/bt2743sup1.cif

Structure factors: contains datablocks I. DOI: 10.1107/S1600536808021363/bt2743Isup2.hkl

Additional supplementary materials:  crystallographic information; 3D view; checkCIF report

## Figures and Tables

**Table 1:** Hydrogen-bond geometry (Å, °)

*D*—H⋯*A*	*D*—H	H⋯*A*	*D*⋯*A*	*D*—H⋯*A*
C5—H5⋯O2^i^	0.95	2.51	3.2726 (13)	137
C13—H13*A*⋯O5^i^	0.98	2.58	3.3715 (15)	138

Symmetry code: (i) 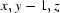.

**Fig. 1.**
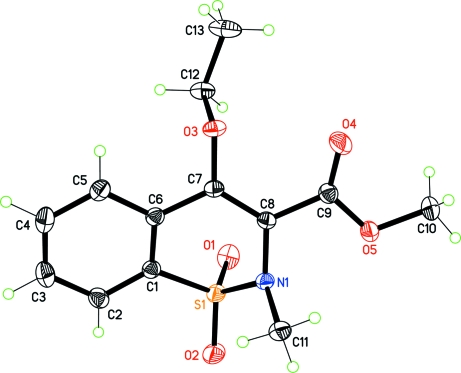
The molecular structure of (I), with displacement ellipsoids at the 50% probability level.

**Fig. 2.**
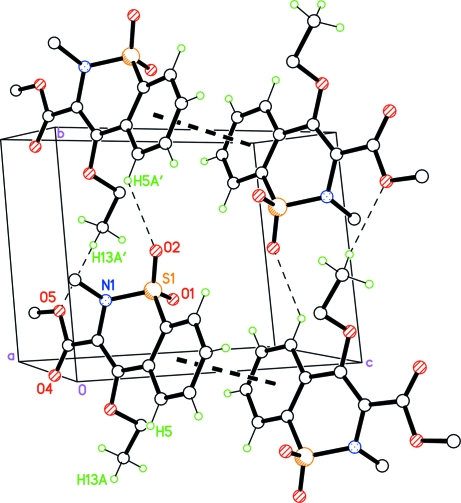
Packing plot showing the two unique weak H-bonds (thin dashed lines) and π-π interactions (thick dashed lines).

## supplementary crystallographic information

### Comment

1,2-Benzothiazine 1,1-dioxides are heterocyclic compounds with numerous types of biological activity reported. For example, some are found useful as medicaments in the treatment of inflammation and rheumatoid arthritis (Lombardino *et al.*, 1971). Other 1,2-benzothiazine 1,1-dioxides exhibit hyperlipidemic, anti-bacterial and Calpain I inhibition activities while they have also been found useful as endothelin receptor antagonists (Bihovsky *et al.*, 2004). In continuation to our ongoing work on the synthesis of benzothiazine 1,1-dioxides (Zia-ur-Rehman *et al.*, 2005, 2006, 2007; Ahmad *et al.*, 2008), we herein report the synthesis and crystal structure of the title compound, (I).

In (I), the thiazine ring exhibits a distorted half-chair conformation with S1/C1/C6/C7 relatively planar (within +/- 0.0336 (6) Å) and N1 showing significant deviation from this plane due to its pyramidal geometry with the methyl group pointing approximately perpendicular to the plane. Compared with related molecules having no substitution at O3 [1.352 (9) Å; Golič *et al.*, 1987; 1.339 (15) Å; Kojić-Prodić & Rużić-Toroš, 1982; 1.350 (9) Å; Reck *et al.*, 1988; 1.336 (2) Å; Fabiola *et al.*, 1998], C9—O4 in (I) is found to be shorter due to the absence of hydrogen bonding at O4. Each molecule of (I) is linked to its neighbours through inter-molecular C-H···S and C-H···O interactions giving rise to chains of molecules parallel to *b* (Fig. 2). Additionally, the molecules in   are linked into centro-symmetric dimers by means of   π-π interactions. The ring centroids are separated by 3.619 Å, while the closest C···C contacts are between C2 and C4'', separated by 3.514 Å (symmetry operator -*x*, -*y*, 1 - *z*).

### Experimental

A mixture of methyl 4-hydroxy-2-methyl-2*H*-1,2-benzothiazine-3-carboxylate 1,1-dioxide (1.33 g; 5.0 mmoles), ethyl iodide (3.90 g; 25.0 mmoles), anhydrous potassium carbonate (10.0 g) and acetonitrile (100 ml) was stirred and refluxed for a period of 7 h. After removal of acetonitrile and excess methyl iodide under vacuum, chloroform (30 ml) was added and the resultant mixture was filtered. The filtrate was washed with water to remove potassium carbonate, dried with anhydrous sodium sulfate and filtered. Slow evaporation of the solvent afforded the crystalline product. Yield:1.26 g; 77.8%; m.p.147°C.

### Refinement

H atoms were placed in geometric positions (C—H distance = 0.95 Å for aryl-H and mthylene-H; 0.98 Å for methyl-H) using a riding model with rotational freedom in the case of the methyl group. *U* values were set to 1.2*U*_eq_ of the carrier atom (1.5*U* for methyl-H).

### Crystal data

**Table:**

C_13_H_15_NO_5_S	*Z* = 2
*M**_r_* = 297.32	*F*_000_ = 312
Triclinic, *P*1	*D*_x_ = 1.458 Mg m^−^^3^
Hall symbol: -P 1	Mo *K*α radiation λ = 0.71073 Å
*a* = 7.9810 (4) Å	Cell parameters from 4878 reflections
*b* = 8.1215 (4) Å	θ = 2.6–30.5º
*c* = 10.8173 (6) Å	µ = 0.26 mm^−^^1^
α = 89.4783 (7)º	*T* = 150 (2) K
β = 79.5124 (8)º	Block, colourless
γ = 79.3434 (7)º	0.57 × 0.17 × 0.10 mm
*V* = 677.33 (6) Å^3^	

### Data collection

**Table:**

Bruker APEXII CCD diffractometer	4069 independent reflections
Radiation source: fine-focus sealed tube	3678 reflections with *I* > 2σ(*I*)
Monochromator: graphite	*R*_int_ = 0.014
*T* = 150(2) K	θ_max_ = 30.6º
ω rotation with narrow frames scans	θ_min_ = 2.6º
Absorption correction: multi-scan(SADABS; Sheldrick, 2007)	*h* = −11→11
*T*_min_ = 0.867, *T*_max_ = 0.975	*k* = −11→11
8153 measured reflections	*l* = −15→15

### Refinement

**Table:**

Refinement on *F*^2^	Secondary atom site location: difference Fourier map
Least-squares matrix: full	Hydrogen site location: inferred from neighbouring sites
*R*[*F*^2^ > 2σ(*F*^2^)] = 0.033	H-atom parameters constrained
*wR*(*F*^2^) = 0.096	*w* = 1/[σ^2^(*F*_o_^2^) + (0.0555*P*)^2^ + 0.1549*P*] where *P* = (*F*_o_^2^ + 2*F*_c_^2^)/3
*S* = 1.07	(Δ/σ)_max_ < 0.001
4069 reflections	Δρ_max_ = 0.47 e Å^−^^3^
184 parameters	Δρ_min_ = −0.34 e Å^−^^3^
Primary atom site location: structure-invariant direct methods	Extinction correction: none

### Special details

**Table:**

Geometry. All e.s.d.'s (except the e.s.d. in the dihedral angle between two l.s. planes) are estimated using the full covariance matrix. The cell e.s.d.'s are taken into account individually in the estimation of e.s.d.'s in distances, angles and torsion angles; correlations between e.s.d.'s in cell parameters are only used when they are defined by crystal symmetry. An approximate (isotropic) treatment of cell e.s.d.'s is used for estimating e.s.d.'s involving l.s. planes.
Refinement. Refinement of *F*^2^ against ALL reflections. The weighted *R*-factor *wR* and goodness of fit *S* are based on *F*^2^, conventional *R*-factors *R* are based on *F*, with *F* set to zero for negative *F*^2^. The threshold expression of *F*^2^ > σ(*F*^2^) is used only for calculating *R*-factors(gt) *etc*. and is not relevant to the choice of reflections for refinement. *R*-factors based on *F*^2^ are statistically about twice as large as those based on *F*, and *R*- factors based on ALL data will be even larger.

### Fractional atomic coordinates and isotropic or equivalent isotropic displacement parameters (Å^2^)

**Table:**

	*x*	*y*	*z*	*U*_iso_*/*U*_eq_	
N1	0.37350 (11)	0.28852 (10)	0.19858 (8)	0.01854 (16)	
C11	0.32384 (14)	0.38127 (14)	0.08880 (10)	0.0245 (2)	
H11A	0.4141	0.3475	0.0145	0.037*	
H11B	0.3107	0.5019	0.1052	0.037*	
H11C	0.2137	0.3560	0.0738	0.037*	
S1	0.24645 (3)	0.32872 (3)	0.33583 (2)	0.02022 (8)	
O1	0.34949 (12)	0.28319 (11)	0.43054 (8)	0.02893 (18)	
O2	0.15077 (12)	0.49632 (10)	0.33569 (9)	0.0320 (2)	
C1	0.10979 (13)	0.18357 (12)	0.33574 (9)	0.01836 (18)	
C2	−0.06595 (13)	0.22200 (14)	0.38733 (10)	0.0230 (2)	
H2	−0.1177	0.3311	0.4207	0.028*	
C3	−0.16426 (14)	0.09717 (16)	0.38904 (10)	0.0268 (2)	
H3	−0.2851	0.1213	0.4223	0.032*	
C4	−0.08623 (15)	−0.06293 (15)	0.34218 (10)	0.0259 (2)	
H4	−0.1538	−0.1483	0.3463	0.031*	
C5	0.08906 (14)	−0.09988 (13)	0.28949 (10)	0.02141 (19)	
H5	0.1406	−0.2099	0.2580	0.026*	
C6	0.18987 (12)	0.02506 (12)	0.28280 (9)	0.01735 (17)	
C7	0.37251 (12)	−0.00786 (12)	0.21908 (9)	0.01697 (17)	
O3	0.45163 (10)	−0.17111 (9)	0.19875 (7)	0.02058 (15)	
C12	0.49827 (15)	−0.25298 (13)	0.31109 (10)	0.0239 (2)	
H12A	0.3954	−0.2867	0.3633	0.029*	
H12B	0.5423	−0.1753	0.3617	0.029*	
C13	0.63614 (18)	−0.40461 (15)	0.27026 (14)	0.0356 (3)	
H13A	0.5911	−0.4810	0.2207	0.053*	
H13B	0.6698	−0.4619	0.3446	0.053*	
H13C	0.7375	−0.3699	0.2189	0.053*	
C8	0.45807 (12)	0.11773 (12)	0.17707 (9)	0.01727 (17)	
C9	0.64295 (13)	0.08859 (13)	0.11182 (9)	0.01947 (18)	
O4	0.72988 (11)	−0.04393 (11)	0.07302 (9)	0.0333 (2)	
O5	0.70186 (10)	0.23360 (10)	0.10291 (8)	0.02397 (16)	
C10	0.88320 (14)	0.21929 (15)	0.04958 (11)	0.0264 (2)	
H10A	0.9526	0.1425	0.0994	0.040*	
H10B	0.9156	0.3299	0.0503	0.040*	
H10C	0.9047	0.1758	−0.0372	0.040*	

### Atomic displacement parameters (Å^2^)

**Table:**

	*U*^11^	*U*^22^	*U*^33^	*U*^12^	*U*^13^	*U*^23^
N1	0.0194 (4)	0.0142 (3)	0.0198 (4)	−0.0011 (3)	0.0003 (3)	0.0009 (3)
C11	0.0246 (5)	0.0219 (5)	0.0254 (5)	−0.0017 (4)	−0.0033 (4)	0.0062 (4)
S1	0.02258 (13)	0.01530 (12)	0.02126 (13)	−0.00367 (9)	0.00029 (9)	−0.00292 (8)
O1	0.0339 (4)	0.0336 (4)	0.0221 (4)	−0.0119 (3)	−0.0065 (3)	−0.0039 (3)
O2	0.0330 (4)	0.0152 (4)	0.0405 (5)	−0.0009 (3)	0.0084 (4)	−0.0039 (3)
C1	0.0194 (4)	0.0180 (4)	0.0173 (4)	−0.0033 (3)	−0.0025 (3)	0.0017 (3)
C2	0.0203 (4)	0.0257 (5)	0.0204 (4)	−0.0009 (4)	−0.0006 (4)	0.0008 (4)
C3	0.0194 (4)	0.0379 (6)	0.0230 (5)	−0.0080 (4)	−0.0015 (4)	0.0020 (4)
C4	0.0243 (5)	0.0325 (5)	0.0246 (5)	−0.0137 (4)	−0.0058 (4)	0.0048 (4)
C5	0.0241 (5)	0.0204 (4)	0.0219 (4)	−0.0074 (4)	−0.0067 (4)	0.0027 (4)
C6	0.0184 (4)	0.0171 (4)	0.0169 (4)	−0.0035 (3)	−0.0039 (3)	0.0019 (3)
C7	0.0189 (4)	0.0143 (4)	0.0175 (4)	−0.0017 (3)	−0.0041 (3)	−0.0008 (3)
O3	0.0266 (4)	0.0134 (3)	0.0204 (3)	−0.0003 (3)	−0.0040 (3)	−0.0010 (2)
C12	0.0284 (5)	0.0179 (4)	0.0251 (5)	0.0011 (4)	−0.0094 (4)	0.0004 (4)
C13	0.0396 (7)	0.0210 (5)	0.0446 (7)	0.0095 (5)	−0.0183 (6)	−0.0072 (5)
C8	0.0171 (4)	0.0153 (4)	0.0185 (4)	−0.0014 (3)	−0.0023 (3)	−0.0010 (3)
C9	0.0186 (4)	0.0202 (4)	0.0190 (4)	−0.0033 (3)	−0.0022 (3)	−0.0012 (3)
O4	0.0237 (4)	0.0231 (4)	0.0472 (5)	−0.0014 (3)	0.0062 (4)	−0.0086 (4)
O5	0.0175 (3)	0.0207 (3)	0.0323 (4)	−0.0045 (3)	0.0003 (3)	−0.0009 (3)
C10	0.0176 (4)	0.0304 (5)	0.0299 (5)	−0.0059 (4)	0.0002 (4)	0.0026 (4)

### Geometric parameters (Å, °)

**Table:**

N1—C8	1.4270 (12)	C5—H5	0.9500
N1—C11	1.4762 (13)	C6—C7	1.4713 (13)
N1—S1	1.6363 (9)	C7—C8	1.3599 (13)
C11—H11A	0.9800	C7—O3	1.3602 (11)
C11—H11B	0.9800	O3—C12	1.4546 (12)
C11—H11C	0.9800	C12—C13	1.5014 (15)
S1—O1	1.4318 (9)	C12—H12A	0.9900
S1—O2	1.4324 (8)	C12—H12B	0.9900
S1—C1	1.7479 (10)	C13—H13A	0.9800
C1—C2	1.3901 (14)	C13—H13B	0.9800
C1—C6	1.4035 (13)	C13—H13C	0.9800
C2—C3	1.3900 (16)	C8—C9	1.4920 (13)
C2—H2	0.9500	C9—O4	1.2008 (13)
C3—C4	1.3904 (17)	C9—O5	1.3429 (12)
C3—H3	0.9500	O5—C10	1.4409 (12)
C4—C5	1.3891 (15)	C10—H10A	0.9800
C4—H4	0.9500	C10—H10B	0.9800
C5—C6	1.4006 (14)	C10—H10C	0.9800
			
C8—N1—C11	116.99 (8)	C5—C6—C7	120.92 (9)
C8—N1—S1	114.71 (6)	C1—C6—C7	121.18 (9)
C11—N1—S1	118.75 (7)	C8—C7—O3	120.74 (9)
N1—C11—H11A	109.5	C8—C7—C6	122.18 (9)
N1—C11—H11B	109.5	O3—C7—C6	117.02 (8)
H11A—C11—H11B	109.5	C7—O3—C12	113.59 (7)
N1—C11—H11C	109.5	O3—C12—C13	107.98 (9)
H11A—C11—H11C	109.5	O3—C12—H12A	110.1
H11B—C11—H11C	109.5	C13—C12—H12A	110.1
O1—S1—O2	119.30 (6)	O3—C12—H12B	110.1
O1—S1—N1	107.81 (5)	C13—C12—H12B	110.1
O2—S1—N1	108.08 (5)	H12A—C12—H12B	108.4
O1—S1—C1	108.19 (5)	C12—C13—H13A	109.5
O2—S1—C1	110.56 (5)	C12—C13—H13B	109.5
N1—S1—C1	101.38 (5)	H13A—C13—H13B	109.5
C2—C1—C6	122.35 (9)	C12—C13—H13C	109.5
C2—C1—S1	122.00 (8)	H13A—C13—H13C	109.5
C6—C1—S1	115.63 (7)	H13B—C13—H13C	109.5
C3—C2—C1	118.52 (10)	C7—C8—N1	120.24 (9)
C3—C2—H2	120.7	C7—C8—C9	123.51 (9)
C1—C2—H2	120.7	N1—C8—C9	116.22 (8)
C2—C3—C4	120.16 (10)	O4—C9—O5	123.71 (9)
C2—C3—H3	119.9	O4—C9—C8	126.14 (9)
C4—C3—H3	119.9	O5—C9—C8	110.15 (8)
C5—C4—C3	120.97 (10)	C9—O5—C10	115.19 (8)
C5—C4—H4	119.5	O5—C10—H10A	109.5
C3—C4—H4	119.5	O5—C10—H10B	109.5
C4—C5—C6	120.01 (10)	H10A—C10—H10B	109.5
C4—C5—H5	120.0	O5—C10—H10C	109.5
C6—C5—H5	120.0	H10A—C10—H10C	109.5
C5—C6—C1	117.87 (9)	H10B—C10—H10C	109.5
			
C8—N1—S1—O1	58.91 (8)	S1—C1—C6—C7	−6.73 (12)
C11—N1—S1—O1	−155.79 (8)	C5—C6—C7—C8	159.51 (10)
C8—N1—S1—O2	−170.89 (7)	C1—C6—C7—C8	−18.73 (14)
C11—N1—S1—O2	−25.58 (9)	C5—C6—C7—O3	−17.78 (13)
C8—N1—S1—C1	−54.62 (8)	C1—C6—C7—O3	163.98 (9)
C11—N1—S1—C1	90.69 (8)	C8—C7—O3—C12	106.82 (11)
O1—S1—C1—C2	104.51 (9)	C6—C7—O3—C12	−75.85 (11)
O2—S1—C1—C2	−27.82 (10)	C7—O3—C12—C13	−159.92 (9)
N1—S1—C1—C2	−142.26 (9)	O3—C7—C8—N1	179.78 (8)
O1—S1—C1—C6	−74.15 (9)	C6—C7—C8—N1	2.59 (14)
O2—S1—C1—C6	153.53 (8)	O3—C7—C8—C9	−2.63 (15)
N1—S1—C1—C6	39.09 (8)	C6—C7—C8—C9	−179.82 (9)
C6—C1—C2—C3	1.63 (15)	C11—N1—C8—C7	−107.70 (11)
S1—C1—C2—C3	−176.93 (8)	S1—N1—C8—C7	38.24 (12)
C1—C2—C3—C4	1.25 (16)	C11—N1—C8—C9	74.55 (11)
C2—C3—C4—C5	−2.02 (17)	S1—N1—C8—C9	−139.52 (8)
C3—C4—C5—C6	−0.10 (16)	C7—C8—C9—O4	11.14 (17)
C4—C5—C6—C1	2.85 (15)	N1—C8—C9—O4	−171.18 (10)
C4—C5—C6—C7	−175.45 (9)	C7—C8—C9—O5	−168.23 (9)
C2—C1—C6—C5	−3.67 (15)	N1—C8—C9—O5	9.45 (12)
S1—C1—C6—C5	174.98 (7)	O4—C9—O5—C10	−3.71 (15)
C2—C1—C6—C7	174.62 (9)	C8—C9—O5—C10	175.68 (8)

### Hydrogen-bond geometry (Å, °)

**Table:**

*D*—H···*A*	*D*—H	H···*A*	*D*···*A*	*D*—H···*A*
C5—H5···O2^i^	0.95	2.51	3.2726 (13)	137
C13—H13A···O5^i^	0.98	2.58	3.3715 (15)	138

Symmetry codes: (i) *x*, *y*−1, *z*.
